# Radio Frequency Exposure in Military Contexts: A Narrative Review of Thermal Effects and Safety Considerations

**DOI:** 10.1093/milmed/usaf613

**Published:** 2025-12-25

**Authors:** Mårten Risling, Mattias Günther

**Affiliations:** Department of Neuroscience, Karolinska Institutet, Stockholm, 17177, Sweden; Department of Neuroscience, Karolinska Institutet, Stockholm, 17177, Sweden

## Abstract

**Introduction:**

Radiofrequency (RF) exposure has been extensively studied for potential health risks. Unlike ionizing radiation, RF fields primarily cause thermal health effects, the only established mechanism of biological harm. Regulatory bodies, including the International Commission on Non-Ionizing Radiation Protection (ICNIRP) and the Institute of Electrical and Electronics Engineers (IEEE), set limits to prevent excessive heating. This review examines the relationship between RF exposure, heat generation, and physiological responses, with relevance to civilian and military safety.

**Methods:**

A narrative review of peer-reviewed literature, regulatory reports, and experimental studies was conducted using PubMed, IEEE Xplore, Google Scholar and Scopus. Emphasis was placed on Specific Absorption Rate (SAR) and Cumulative Equivalent Minutes at 43 °C (CEM43). Studies on thermal effects and exposure scenarios were prioritized; speculative non-thermal mechanisms were excluded.

**Results:**

Thermal effects depend on frequency, tissue composition, and environmental conditions. Whole-body SAR limits (≤4 W/kg) generally prevent core temperature increases, but localized heating remains a concern. CEM43 provides a temperature-based metric but is difficult to apply in transient exposures. Penetration depth across NATO frequency bands shows variability because of differences in tissue models and measurement methods. This variability is clinically relevant, as localized heating of the skin, eye, or superficial nerves may occur even when whole-body exposure is within limits.

**Conclusion:**

Current guidelines prevent systemic overheating but may not fully address localized risks. Combining SAR and CEM43 with refined penetration depth data could improve risk assessment. Future work should refine dose–response thresholds and methods for detecting and modeling localized heating, especially under military conditions where thermoregulation may be impaired.

## INTRODUCTION

The potential health risks of exposure to radiofrequency (RF) fields have been extensively studied, and several regulatory bodies publish comprehensive reports. The International Commission on Non-Ionizing Radiation Protection (ICNIRP) provides Guidelines for Limiting Exposure to Electromagnetic Fields (100 kHz-300 GHz).^1^ The IEEE International Committee on Electromagnetic Safety issues safety standards for 0 Hz-300 GHz.^2^ The Swedish Radiation Safety Authority publishes annual updates, such as its 2019:08 report on EMF and health risks.^3^ The European Union’s SCENIHR has also issued an opinion on potential health effects of electromagnetic fields.^4^ From a medical perspective, RF exposure is non-ionizing. Unlike ionizing radiation, which produces cumulative effects over time, RF exposure lacks the energy to dislodge electrons, meaning they do not cause ionization, free radical formation, or chromosomal damage. RF exposure effects are threshold-dependent: below certain levels, no biological harm occurs regardless of duration.^5^ Military RF sources include radar (1-40 GHz), communication systems (3 MHz-3 GHz), and High-Power Microwave (HPM) weapons (hundreds of MHz–tens of GHz). Radar is the most common occupational exposure, although HPM produces intense pulses designed to disable electronics. Millimeter-wave systems (>30 GHz) are also explored for directed-energy applications.^6^ The primary health risk is localized heating, particularly when thermoregulation is impaired. For example, humans exhibit thermoregulatory behavior such as instinctively withdrawing from or moving away when exposed to excessive warmth or discomfort. Current consensus holds that only thermal effects are established as harmful, though clinical guidance on heat physiology in RF contexts remains limited. However, readily accessible information on the physiological effects of heat in humans related to RF remains limited, reducing its practical utility for clinicians. This review examines the relationship between RF exposure, heat generation, and physiological responses, with relevance to safety considerations, primarily in military contexts.

## METHODS

This scoping review followed the PRISMA-ScR framework^7^ to map evidence on the thermal effects of radiofrequency (RF) exposure (Appendix 1), with search strategy and flow diagram in Appendix 2. *Eligibility criteria:* Peer-reviewed studies, regulatory reports, and experimental or review papers (1970–March 2025) addressing RF-induced heating, thermal metrics (SAR or CEM43), or exposure limits were included. Studies proposing non-thermal biological effects were excluded when no quantifiable heating or exposure–response relationship was demonstrated, consistent with the consensus of regulatory bodies (ICNIRP, IEEE C95.1) that only thermal mechanisms are established as causative for health effects from RF exposure. *Information sources and search:* Searches were performed in *PubMed*, *IEEE Xplore*, *Scopus*, and *Google Scholar* using combinations of the terms *“radiofrequency exposure,” “RF-induced heating,” “specific absorption rate,” “CEM43,” “thermal effects,”* and *“military”*. Key regulatory documents from ICNIRP, IEEE, the Swedish Radiation Safety Authority, and SCENIHR were also reviewed. The final search was run on March 15, 2025, and reference lists of included papers were screened. *Selection and data charting:* One reviewer (MG) screened all records and extracted data into Excel, recording study type, frequency, exposure level, SAR/CEM43, tissue type, and reported outcomes; a second reviewer (MR) verified inclusion decisions. *Synthesis:* Results were summarized qualitatively under five themes: (1) RF heating mechanisms, (2) SAR and CEM43 metrics, (3) tissue-specific responses, (4) civilian vs military exposure contexts, and (5) implications for safety standards. No quantitative synthesis was attempted.

## RESULTS

This review focuses on the thermal effects of RF exposure in humans. Non-thermal effects are primarily discussed in relation to High-Power Microwaves (HPM), emitted by military radar or devices targeting electronics. NATO STANAG 2345 outlines safety concerns specific to HPM.^6^ Because HPM pulses are extremely brief, significant heating is unlikely, and Specific Absorption Rate (SAR) has limited value for predicting non-thermal outcomes. Although such mechanisms are under investigation, they fall outside the scope of this review.

### Heat and Human Cells

Early studies in the 1970s showed that below 43 °C and with short exposure times, cell survival curves displayed a shoulder, representing a plateau phase where cells sustained sub-lethal injury but were able to repair damage or develop thermotolerance. At higher temperatures, this compensatory phase disappeared, and cell death increased linearly with exposure time.^8^ Thus, thermal injury depends on both exposure time and temperature. Across 40-55 °C, cell death increases exponentially, with a breakpoint at ∼43 °C marking accelerated mortality.^9^

To quantify tissue heating from RF exposure, two main metrics are used:

1. Cumulative Equivalent Minutes at 43 °C (CEM43): expresses heat load in tissue.
2. Specific Absorption Rate (SAR): measures RF energy absorbed, though not always directly linked to heat generation.

### Cumulative Equivalent Minutes at 43 °C (CEM43)

Sapareto and Dewey introduced CEM43 as a model to calculate a thermal iso-effective dose by converting any time–temperature history into an equivalent heating duration at 43 °C.^10^ In cell culture studies (41-47 °C), survival was assessed by colony assays, viability stains, and apoptosis markers. Results showed an exponential relationship between temperature and exposure time, with higher temperatures causing faster cell death. Normalizing effects to 43 °C enabled comparison of diverse heating conditions. The model was later validated in animal and human tissues, becoming central to hyperthermia therapy, RF safety, and thermal damage prediction.^10^

CEM43 is derived from the Arrhenius equation:


CEM43=∑Δt×R^ (43-T)

where Δt = exposure time, T = tissue temperature (°C), and R adjusts for 1 °C change (≈0.5 for T < 43°C; ≈0.25 for T > 43 °C).^10^ Limitations include: (1) reduced accuracy at extreme temperatures, (2) context-specific definitions of “damage,” (3) difficulty applying to transient RF exposures, (4) reliance on whole-body thresholds, and (5) uncertain extrapolation from animal models.^11^ Despite these caveats, CEM43 remains a practical framework for quantifying thermal dose, though caution is needed when translating results into exposure guidelines.

### Specific Absorption Rate (SAR)

SAR quantifies the rate of RF energy absorbed per unit mass of tissue, expressed in W/kg. Values are typically averaged over the whole body or small tissue volumes (1 g or 10 g). Absorption depends on tissue dielectric properties, which vary by organ, but RF energy deposition does not directly predict temperature rise because of efficient thermoregulation. Vascularized tissues (e.g., brain, muscle, skin) increase blood perfusion up to 1.5× with heating, aiding dissipation. Above 2-3 GHz, conduction dominates cooling. If dissipation were ignored, SAR 10 W/kg would raise tissue temperature ∼0.15 °C/min, reaching +2°C in ∼13 min. In practice, whole-body SAR of ∼4.5 W/kg is required for a 1°C core rise.^12^ Regulatory limits set maximum whole-body SAR at 4 W/kg, based on animal studies where >4 W/kg triggered thermoregulatory responses.^1^^,^^2^^,^^4^^,^^13^^,^^14^ Localized exposures may tolerate higher SAR, as circulation redistributes heat.^15^ Modeling predicts that 0.4 W/kg whole-body SAR increases core temperature only 0.1-0.15 °C after 1 hour. Experimental magnetic resonance imaging (MRI) studies with SAR 0.086-0.95 W/kg for up to 90 min showed no core temperature rise.^16^^,^^17^ In another study, volunteers exposed to 2450 MHz RF at local SAR up to 15.4 W/kg showed no esophageal temperature changes, even at high ambient temperatures.^2^^,^^18^ Thus, although extreme exposures can overwhelm thermoregulation, under regulatory limits SAR-induced heating is effectively controlled. Thermal discomfort and heat sensations provide early behavioral protection before harmful rises occur.

### Comparing SAR and CEM43 for RF Heat Assessment

Specific Absorption Rate (SAR) is the current standard for defining RF exposure limits. Regulatory thresholds for 10 MHz-10 GHz aim to prevent harmful heating, with whole-body SAR capped at 4 W/kg. Comparisons show SAR and CEM43 give broadly consistent results across frequencies, though absorption depth and heating vary with frequency. Both models face limitations because of scarce human data on time–temperature thresholds for adverse effects.

Challenges include:^15^

- SAR requires complex modeling and measurements.
- CEM43 depends on difficult in vivo temperature measurements.
- The link between thermal dose and biological outcomes is not fully defined.
- Temperature-based metrics are more intuitive but harder to standardize.
- SAR is well established, making it the practical default.

Some propose shifting from SAR to temperature-based thresholds, but this would require precise definitions of adverse exposure conditions. The IEEE has mainly raised this in the context of partial-body exposures. Within current ICNIRP and IEEE guidelines, whole-body RF heating is effectively mitigated by thermoregulation. For now, SAR remains the standard metric.^15^

### Civilian Exposures to RF and Military Relevance

MRI provides a key civilian example of RF-induced heating. MRI uses strong static fields (1.5-7 T) and RF waves (64-300 MHz) to generate images, and although safe overall, it can cause localized heating. Reported incidence of skin injury is extremely low (<0.0004%) for fields between 0.2-3.0 T. However, higher field strengths increase RF intensity, raising concern for hotspots. To manage thermal risk, thermal dose metrics such as CEM43 have been proposed.

Two reports set safe thresholds:^9^^,^^19^

1. All individuals: max local temperature ≤39 °C.
2. Compromised thermoregulation: uncontrolled conditions ≤39 °C; controlled TD <2 CEM43 (cumulative equivalent thermal dose of two minutes at 43 °C).
3. Uncompromised thermoregulation: uncontrolled TD <2 CEM43; controlled TD <9 CEM43. For most patients, 9 CEM43 was acceptable; for skin, muscle, fat, and bone, 16 CEM43 appeared safe.^11^ Controlled conditions required immediate medical oversight.

Volunteer MRI studies showed SAR distribution was uneven, with hotspots at joints and muscle attachment points.^20^ Importantly, SAR values do not directly equate to uniform temperature rises. Translating MRI-derived thresholds to military contexts is problematic. Unlike controlled medical settings, military operations rarely ensure immediate intervention. Moreover, operational stressors—high ambient temperature, heavy loads, or immobility—may impair thermoregulation. Thus, although MRI data inform understanding of RF heating, their applicability to military exposure scenarios is limited.

### Tissue-Specific Reactions to Heat

SAR values do not translate to uniform body temperature increases, as thermoregulation (blood flow, sweating) maintains homeostasis. Still, heterogeneous absorption creates hotspots, which may drive acute symptoms in heat-sensitive tissues more than core temperature rise. Monitoring thermal dose via CEM43 helps quantify risk.

A review by van Rhoon et al.^1^ summarized thresholds across 31 tissues in 11 species:

- Bone: Damage threshold ∼16 min CEM43, based on rabbit tibia studies.
- Skin: Acute/chronic damage above 41 min CEM43; necrosis above 288 min. Human studies confirmed erythema and transient sensitivity changes after 112 min CEM43.
- Brain: In monkeys, lesions occurred above 280 min CEM43; 50% necrosis probability estimated at 192 min, none below 90 min.
- Muscle: Minor damage at 41 min CEM43; hemorrhage/necrosis above 80 min.
- Fat: Delayed adipocyte death observed after 15-60 min CEM43 exposures at 43-45 °C.

Hotspots are particularly important during short exposures, when heat dissipation is limited, and at higher frequencies where penetration depth decreases.^1^ Neural tissue may be especially vulnerable: heating at boundaries can cause mechanical stress. The microwave auditory effect may be a thermoelastic response to pulsed fields, although this needs to be verified.^21^^,^^22^

### Temperature Thresholds and Human Effects

The body maintains internal temperatures between 36 and 38 °C, with natural fluctuations from 35.5 to 40 °C because of environment, circadian rhythm, activity, hormones, or substances. Heat tolerance varies by age, sweating capacity, cardiovascular and respiratory function, fat levels, nutrition, pH balance and illness, including fever.^23^ Basal metabolic rate (BMR) averages 1-1.5 W/kg (1200-1500 kcal/day), rising to 2.5 W/kg when climbing stairs, >18 W/kg during sprinting, and up to 30 W/kg in elite athletes, producing heat that must be dissipated to avoid hyperthermia.^24^ A distinction exists between biological and health effects: many physiological changes are tolerated or adapted to without harm. Health effects emerge only when compensatory mechanisms fail.^25^ When core temperature exceeds 40 °C, heat stress and stroke may occur, causing delirium, seizures, coma, or death. Heat stress also impairs motor and cognitive performance. Studies suggest acute RF exposure can disrupt behavior when body temperature rises above 41 °C, with whole-body SAR values of 3.2-8.4 W/kg and power densities of 8-140 mW/cm^2^, effects considered primarily thermal.^25^ Environmental and operational factors can increase susceptibility. For example, dermal burn thresholds in rats at 50 °C varied by applied pressure: 3 min at 28 mmHg (384 CEM43), 2 min at 49 mmHg (256 CEM43), versus 10 min without pressure (1280 CEM43).^26^ In this context, applied pressure refers to localized mechanical pressure exerted on the skin surface. These findings underscore how compromised physiology or stressors like load carriage may lower heat tolerance in combat and extreme environments.

### Penetration of RF Radiation

RF penetration into the body depends on frequency, tissue composition (water, fat, protein, salt), and waveform (Table 1). Continuous waves deposit more energy than pulsed waves. Penetration depth is defined as the point where field amplitude falls to 37% of its original value.^27^ Higher frequencies penetrate less deeply, with absorption concentrated at the surface. ICNIRP notes that above 6 GHz, penetration is confined to superficial tissues: ∼1 cm at 6 GHz and ∼0.4 mm at 300 GHz.^1^ At these frequencies, SAR is no longer a reliable measure of heating; power density at the skin surface provides a better estimate.^28^^,^^29^ Pain perception, triggered by absolute skin temperature, serves as a protective mechanism—individuals exposed to millimeter waves (mm-waves) are likely to withdraw before injury occurs.^30^^,^^31^ Gabriel’s review of dielectric properties confirmed that penetration depth decreases as frequency rises. Low frequencies (<1 GHz) penetrate several centimeters (e.g., ∼4 cm at 1 GHz, exposing ∼21% of body mass), whereas higher frequencies (e.g., 10 GHz) penetrate only ∼0.5 cm (<3% of body mass).^30^^,^^32^ High-water-content tissues (muscle, brain, blood) absorb RF more strongly than low-water-content tissues (fat, bone), limiting penetration depth. Localized “hot spots” complicate predictions, as uneven heating may occur. Deep tissues like fat and muscle can be warmed indirectly via thermal diffusion from the skin, influenced by fat thickness and blood perfusion. Beam geometry also matters: broad RF beams produce higher temperatures than narrow beams at the same power density. Clothing generally has little effect on mm-wave attenuation, though moisture increases absorption.^2^

**Table 1.** RF Penetration into the Human Body

NATO frequency band	Frequency range	Penetrance (mm)		
A	0-250 MHz			
B	250-500 MHz			
C	500-1000 MHz			
D	1-2 GHz	9.4	4.05	
E	2-3 GHz		2.46	
F	3-4 GHz			
G	4-6 GHz		1.66	
H	6-8 GHz			8.1
I	8-10 GHz	1.9	0.65	
J	10-20 GHz		0.46	3.9
K	20-40 GHz	0.54	0.16	9.2
L	40-60 GHz			
M	60-100 GHz			4.9
N	100-200 GHz	0.7		3.5
O	100-200 GHz			
Reference		^28^	^38^	^27^

### Region-Specific Considerations

ICNIRP and NATO STANAG 2345 guidelines aim to minimize systemic heating risks, but some regions may be more vulnerable because of limited heat dissipation. The cornea and lens are considered especially susceptible to RF heating.^33^^,^^34^ Another phenomenon is microwave hearing, described as clicking sounds when entering or leaving an RF field, likely caused by thermoelastic expansion in the cochlear basilar membrane.^21^ Current evidence suggests no permanent auditory effects. By contrast, ocular effects remain of greater concern, particularly the debated link between RF exposure and cataract formation.

### Heat-Related Signs and Symptoms of RF Overexposure

RF exposure can generate heat, but civilian guidelines remain far below hazardous levels.^1^^,^^2^^,^^4^ Above 6 GHz, absorption is limited to surface tissues.^1^ Because thermoregulatory mechanisms are usually effective, most overexposures cause no symptoms; biological effects do not automatically equate to health effects. Even exposures above safety limits often result in no detectable harm.^5^ When compensatory mechanisms are overwhelmed, RF heat may raise core temperature or cause localized effects. Deep regional hyperthermia is perceived as pressure, pain, or discomfort.^35^ The most common symptom is warmth in the exposed region, progressing to pain if exposure continues. Typically, individuals withdraw instinctively, limiting exposure. Localized “hot spots” can form when metals concentrate electromagnetic fields, producing discomfort or burns. Jewelry, orthopedic or dental implants, and other metallic objects have been implicated.^5^ Because RF radiation is invisible and lacks biological markers, suspected overexposure may also trigger anxiety reactions. NATO STANAG 2345 provides standardized medical protocols for examination and evaluation in such cases.

### Military Context and Theoretical Exposure Examples

Operational environments introduce several factors that may alter thermoregulatory capacity and increase susceptibility to RF-induced heat stress. In military settings, personnel may be exposed to high ambient temperatures, heavy physical workloads, dehydration, protective clothing, and limited opportunity for behavioral cooling, all of which reduce heat dissipation efficiency. Under such conditions, even exposures within current SAR limits could theoretically produce localized or cumulative thermal strain. For example, proximity to high-power radar arrays or high-gain communication antennas mounted on vehicles could result in transient surface heating, particularly when reflective metallic structures amplify local fields. Similarly, enclosed or armored environments may impair convective heat loss and promote accumulation of RF energy. Although existing guidelines provide wide safety margins, these scenarios highlight the need for continued refinement of exposure models that integrate environmental stressors, operational posture, and physiological limits. Future work should therefore focus on validating risk thresholds under combined RF and environmental heat loads representative of real-world military operations.

For military medical providers, awareness of the clinical presentation and management of suspected RF overexposure is critical. The recent U.S. DoD Joint Trauma System Clinical Practice Guideline for Suspected RF Electromagnetic Field Overexposure provides a structured framework for evaluation and triage.^36^ It emphasizes immediate removal from exposure, assessment of localized burns or sensory disturbances, monitoring for systemic heat stress, and documentation of exposure circumstances. Incorporating these recommendations into military training and operational medical planning could enhance readiness to recognize and manage RF-related incidents, particularly in environments where thermoregulatory strain and equipment-induced reflections may increase risk. Anxiety reactions following suspected RF overexposure have been described in military and occupational settings, emphasizing the importance of clinical evaluation even when objective injury is absent.^37^

## CONCLUSIONS

Current RF safety guidelines, primarily based on SAR limits, effectively prevent excessive heat accumulation in the body, but localized heating remains a key concern, particularly in environments where thermoregulation is compromised. Although the body dissipates heat efficiently through perfusion and sweating, tissue-specific heating thresholds vary, and prolonged or intense exposure can create hotspots with potential for thermal damage. Metrics like CEM43 offer a temperature-based assessment of risk, but further refinement is needed for real-world applications. Future research should focus on integrating SAR and thermal dose models to better predict heat-related risks, especially high-stress operational settings.

## Supplementary Material

usaf613_Supplementary_Data

## Data Availability

Data sharing is not applicable to this article, but supporting materials are available from the corresponding author on reasonable request.

## INSTITUTIONAL CLEARANCE

Does not apply.
