# Supplementary material for: Radio Frequency Exposure in Military Contexts: A Narrative Review of Thermal Effects and Safety Considerations
Source: Mil Med. 2025 Dec 25;191(9-10):e1858–63. doi: 10.1093/milmed/usaf613 (PMC13542620; doi:10.1093/milmed/usaf613)
Supplement: Supplementary file 1 [file usaf613_supplementary_data.zip › Appendix2.pdf]

Full Electronic Search Strategy

Below is the complete search syntax for PubMed.Database: PubMed
Date last searched: 15 March 2025
Coverage period: 1970–March 2025
Search limits: English language, peer-reviewed publications, humans and animal studies.
Search string:

(("radio frequency"[MeSH Terms] OR "radiofrequency"[All Fields] OR "RF exposure"[All Fields])

AND ("heating"[All Fields] OR "thermal effects"[All Fields] OR "specific absorption rate"[All Fields]

OR "SAR"[All Fields] OR "CEM43"[All Fields] OR "thermal dose"[All Fields])

AND ("safety"[All Fields] OR "guidelines"[All Fields] OR "regulations"[All Fields]

OR "occupational exposure"[All Fields] OR "military"[All Fields] OR "defense"[All Fields])

NOT ("non-thermal"[All Fields] OR "oxidative stress"[All Fields] OR "DNA damage"[All Fields]))

Equivalent term sets were used for:

- IEEE Xplore: ("radiofrequency" OR "RF exposure") AND ("SAR" OR "CEM43" OR "thermal effects") AND ("safety" OR "military")
- Scopus: TITLE-ABS-KEY("radiofrequency exposure" AND ("SAR" OR "CEM43") AND ("thermal" OR "heating") AND ("military" OR "occupational safety"))
- Google Scholar: "radiofrequency heating military" OR "RF exposure thermal dose CEM43"

Additional grey literature was identified from:

- ICNIRP Guidelines (2020)
- IEEE C95.1 Standard (2019)
- Swedish Radiation Safety Authority (SSM 2019:08)
- NATO STANAG 2345
- U.S. DoD Joint Trauma System CPG (2024)

Duplicates were removed manually, and references of included papers were screened to identify additional relevant sources.

Selection Process and Data Charting

- Screening:
  One reviewer (MG) screened all retrieved titles and abstracts; a second reviewer (MR) verified eligibility.
- Inclusion Criteria:
  Peer-reviewed studies, regulatory documents, and experimental or review papers addressing RF-induced heating, thermal metrics (SAR or CEM43), or exposure limits.

- Exclusion Criteria:
  Studies proposing “non-thermal” biological effects without quantifiable heating or exposure–response relationships, per ICNIRP and IEEE consensus
- Data Extraction:
  Data were charted in Excel.

PRISMA-ScR Flow Diagram

Identification

Records identified through database searches (n = 182)

PubMed (n = 86) | IEEE Xplore (n = 33) | Scopus (n = 43) | Google Scholar (n = 20)

Additional records identified through reference lists and regulatory reports (n = 7)

Screening

Duplicates removed (n = 26)

Records screened by title/abstract (n = 163)

Records excluded (n = 113)

Eligibility

Full-text articles assessed for eligibility (n = 50)

Full-text articles excluded (n = 12)

Included

Studies included in qualitative synthesis (n = 38)
